# Counter-Rotate Technique Is Substantial for Correcting Thoracolumbar/Lumbar Curvature in AIS Patients with Thoracic Scoliosis

**DOI:** 10.3390/jcm14030706

**Published:** 2025-01-22

**Authors:** Shoji Seki, Peter O. Newton, Hiroto Makino, Hayato Futakawa, Katsuhiko Kamei, Yushi Yashima, Yoshiharu Kawaguchi

**Affiliations:** 1 Department of Orthopaedic Surgery, Faculty of Medicine, University of Toyama, 2630 Sugitani, Toyama 930-0194, Japan; hiroto@med.u-toyama.ac.jp (H.M.); hayato83@med.u-toyama.ac.jp (H.F.); yushi@med.u-toyama.ac.jp (Y.Y.); zenji@med.u-toyama.ac.jp (Y.K.); 2 Department of Orthopedics, Rady Children’s Hospital-San Diego, 3020 Children’s Way, San Diego, CA 92123, USA; pnewton@rchsd.org; 3 Department of Orthopaedic Surgery, Toyama Red Cross Hospital, 2-1-58 Ushijima Honmachi, Toyama 930-8562, Japan; k.kamei9786@gmail.com

**Keywords:** adolescent idiopathic scoliosis, posterior spinal fusion, counter-rotate technique, thoracic scoliosis, thoracolumbar scoliosis, lumbar curvature

## Abstract

**Background/Objectives**. Correction of thoracolumbar/lumbar curvature in adolescent idiopathic scoliosis (AIS) patients with Lenke 1-2 B and C is still controversial, with regard to extension of the caudal side to the lowest instrumented vertebra (LIV) and method of correction. We assessed the association between change in thoracolumbar/lumbar curvature after surgery with counterrotate technique (CRT) and clinical factors in 45 thoracic AIS patients. **Methods**. Forty-five AIS patients (mean follow-up 5.1 y, age 15 y, Type B: 28, Type C: 17) were analyzed. Posterior spinal fusion was performed by the placing of segmental uni-planar screws, concave rod rotation, differential rod countering, and segmental CRT. Association between change in thoracolumbar/lumbar curvature after surgery with counter-rotate technique and clinical factors was analyzed in 45 thoracic AIS patients. **Results**. Mean main thoracic Cobb angle was 52°, and mean thoracolumbar/lumbar curvature Cobb angle was 35°. Postoperative thoracolumbar/lumbar Cobb was 10.1, and final follow-up was 8.2. Multi logistic regression analysis of change in thoracolumbar/lumbar Cobb after surgery was performed. Age (*p* < 0.05), Risser sign (*p* < 0.05), and postoperative thoracolumbar/lumbar Cobb (*p* < 0.0001) were significantly associated with a change in Cobb angle. **Conclusions**. Correction of thoracolumbar/lumbar curvature using CRT showed significant improvement of thoracolumbar/lumbar curvature, LIV tilting angle, and vertebral rotation. Postoperative thoracolumbar/lumbar Cobb angle (1st erect) was the most significant factor associated with deterioration of thoracolumbar/lumbar curvature after surgery. Subsequent rotational correction of thoracolumbar/lumbar curvature is likely to prevent the deterioration of thoracolumbar/lumbar Cobb after surgery.

## 1. Introduction

Adolescent idiopathic thoracic scoliosis (AIS) causes the three-dimensional deformation of the spine and thorax during their growth spurt. In the long-term natural course of untreated idiopathic scoliosis, scoliosis of 45–50 degrees will continue to progress even after bone maturation is complete [1]. Moreover, severe untreated spinal deformity can cause severe trunk deformity, decreased lung function, and lower-back pain. Surgical intervention with spinal instrumentation and fusion can correct spinal deformity and prevent curve progression, and most studies have shown satisfactory long-term radiographic and clinical results [2,3]. The ultimate goal of treatment is to balance the spine in all aspects. However, there is still no consensus on how to correct the thoracolumbar curvature in AIS patients with a thoracic curve presenting as Lenke 1-2 B and C [3,4]. To date, various methods have been reported for treating Lenke 1-2 B and C, including selective thoracic fusion, which does not actively correct the thoracolumbar/lumbar curvature [5], LIV extension [6], and methods that fix the lumbar curvature [7]. Each method has its advantages and disadvantages, and there have been reported cases in which additional surgery was required due to the long-term deterioration of the lumbar curve if the thoracolumbar/lumbar curve remained [3]. In recent years, methods such as spinal tethering to correct the lumbar curve have also been reported, but the long-term results remain unknown [8,9]. Several long-term follow-up studies of postoperative AIS patients have shown that spinal fusion to the middle or lower lumbar spine may have adverse effects, including unfixed lumbar intervertebral disc degeneration below the fixed intervertebral space [3,10,11,12]. On the other hand, some reports on the long-term outcome of selective thoracic fusion show that the lumbar curve is good in the long term, even if there is a well-balanced lumbar curve [13,14]. In any case, there are a few reports of long-term outcomes associated with the deterioration of the residual thoracolumbar/lumbar curvature, which can lead to lower-back pain and nerve injury in the lower limbs [15]. It has also been reported that the larger the residual curve, the lower the ODI and the greater its interference with daily life [16,17,18]. Given these facts, it is considered necessary to have a well-balanced lumbar spine, with no fixation extending to the lower lumbar spine, and no residual lumbar curve.

Based on the above, it is considered desirable to correct the thoracolumbar/lumbar curvature as much as possible to the Lenke 1-2 B and C curve. In addition, there are no reports that we have been able to find on the Lenke type 1-2 B and C curve that describe in detail the method of correcting the lumbar curve or the long-term results. There are also reports that if the thoracolumbar/lumbar curvature is fixed by the excessive extension of the LIV, lower-back pain will worsen due to reduced lumbar mobility and the associated worsening of adjacent intervertebral disorders [19]. For these reasons, we have tried to avoid extending fixation to the lower lumbar spine while aiming for selective thoracic fusion. Therefore, we have extended the range of fixation to one vertebra below the stable vertebra (SV), and have tried to correct the lumbar curve adequately using the counter-rotate technique (CRT), which corrects the lumbar rotation as much as possible using that vertebra. The purpose of this study was to evaluate the effectiveness of CRT to correct lumbar curves in patients with Lenke 1-2 B and C curves.

## 2. Materials and Methods

### 2.1. Subjects

Of 121 patients with Lenke type 1 or 2 AIS, 45 patients with Lumbar Modifier B or C who underwent CRT and were followed-up with for more than 2 years after surgery (mean follow-up period 5.1 years, mean age 15 years) were included in the study. According to the classification of Lenke et al. [4], Lumbar Modifier B and C patients were numbered 28 and 17, respectively. All patients had been treated with a Boston brace as a conservative treatment prior to surgery. Patients with a Cobb angle of 20–25 degrees or greater and a Risser grade of 3 or less wore the brace at night for at least six months. If progression of the scoliosis was still observed, surgical treatment was recommended. All posterior corrective spinal fusions were performed by one spine surgeon specializing in scoliosis (S.S.). Exclusion criteria in this study included a body mass index above the range of 2 standard deviations, open-chest or other cardiac surgery, severe psychiatric disorders, neuromuscular diseases including syringomyelia cordata, and patients undergoing hormonal treatment. Patients were evaluated by X-ray and CT for preoperative and postoperative screw insertion and apical spinal rotation. X-ray data were analyzed preoperatively and at least 2 years postoperatively. Thereafter, the patients were checked by X-ray every year until the final follow-up. This study was approved in advance by the Ethics Review Committee at the University of Toyama (No. 28-108). In addition, informed consent for participation in the study and consent for the surgery were obtained prior to surgery.

### 2.2. Surgical Procedure

#### Correction of Thoracolumbar/Lumbar Curve Using CRT

**Figure 1:**
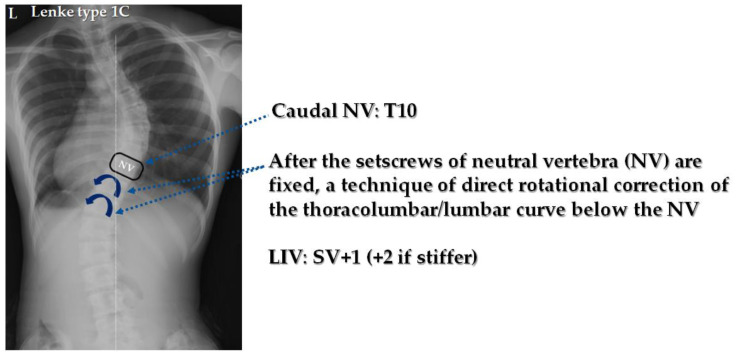
After concave rod rotation followed by differential rod contouring (DRC), the setscrews of the neutral vertebra (NV) were fixed for the concave and convex rods. To correct the lumbar curve, the vertebrae below the NV were aggressively applied with corrective force for each vertebral rotation.

**Figure 2:**
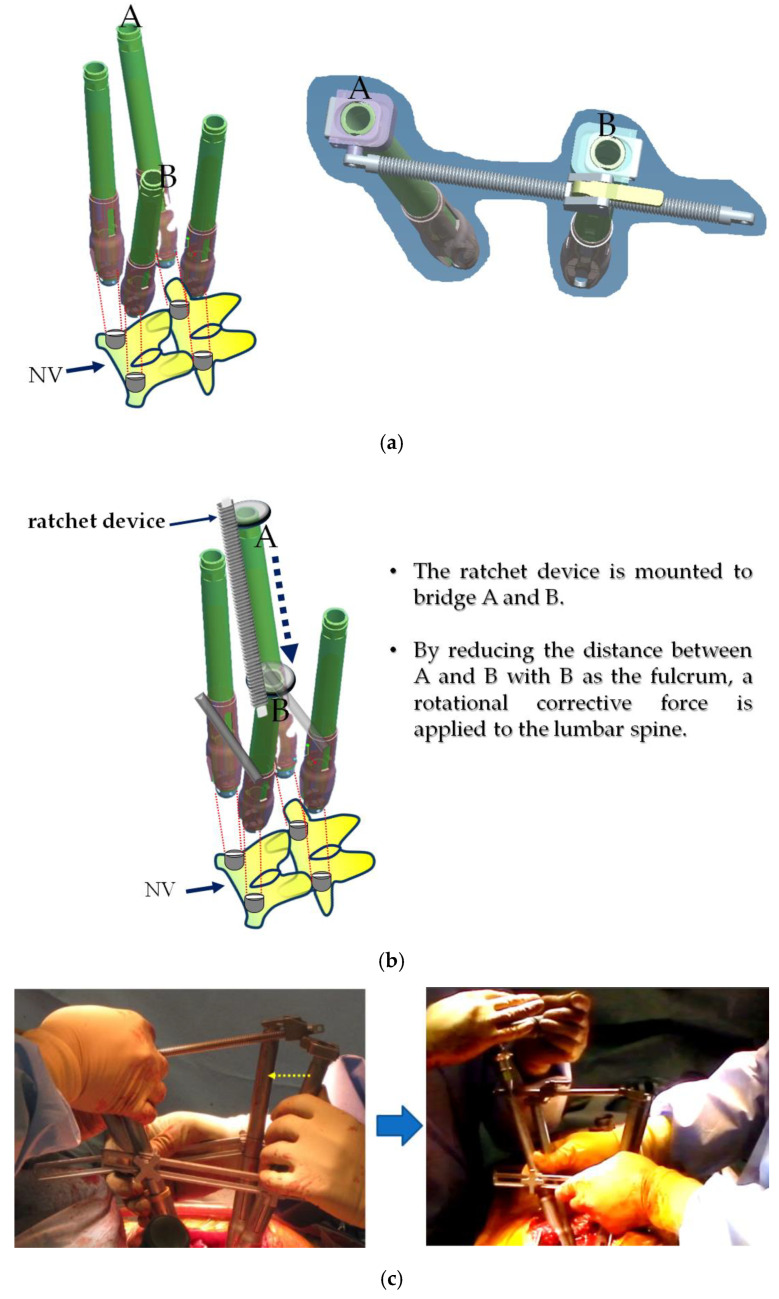
We developed a novel ratchet device for the counter-rotate technique (CRT). The ratchet device is attached to the lumbar region to be corrected. A is connected to A and B is connected to B to perform CRT (**a**). Ratchet devices connect to the bridges A and B. With B as the fulcrum, the distance between A and B is reduced. This applies a rotational corrective force to the lumbar vertebrae (**b**). Due to the ratchet structure, once corrective force is applied, it will not return to the original position even if the hand is released, and the corrective force will be maintained. The arrows in the figure on the left indicate the direction in which the stick is moved. Photographs show intraoperative correction methods (**c**). The photo on the left is before CRT. The yellow dotted arrows indicate that aligning the stick applies a rotational corrective force to the vertebral body. The right photo shows after the correction. Both sticks are aligned to correct the rotation, and the setscrews are fastened.

The corrective technique with CRT (shown in Figure 1) was performed in all patients with lumbar modifier B or C. A skin incision was made in the posterior midline, exposing the posterior spine. After exposing the posterior elements of the spine, a segmental uni-planar screw was inserted using a free-hand technique. Next, the facet joint and its articular cartilage in the range of fixation were removed, and a Ponte osteotomy [20] was performed around periapical region. Next, rod rotation (RR) was performed on the concave side of the scoliosis. After the concave RR was performed, differential rod contouring (DRC) was performed on the convex side as previously described [21]. The rod material was CoCr with a diameter of 5.5 mm. Next, CRT was performed with each vertebra, from the most caudal neutral vertebra (NV) to the lowest instrumented vertebra (LIV), for correcting the thoracolumbar/lumbar curve. The actual technique is similar to direct vertebral rotation (DVR) [22], but is characterized by the use of a novel ratchet device to apply a rotational corrective force indirectly to the lumbar spine curve below the neutral vertebra curvature, using the opposite rotation of the thoracic spine curve (Figure 2). This device can apply a stronger and more direct rotational corrective force to the lower vertebrae, and the ratchet structure prevents the loss of corrective force. After the correction of the scoliosis, the coronal balance of the spine was confirmed by fluoroscopy and X-ray. All patients who underwent surgery were evaluated intraoperatively with spinal cord monitoring using motor and somatosensory evoked potentials. 

### 2.3. Radiographical and CT Imaging Evaluation

All patients who participated in this study were evaluated by X-ray and CT scanning before and after surgery. CT scanning was used pre- and postoperatively to confirm the planned size of the screw insertion and whether it was inserted in the correct position. CT scanning was also used to measure the vertebral rotation of the thoracolumbar/lumbar curve before and after surgery. To ensure the accuracy of the angular measurements of the X-ray images, we used an image analyzer, Synapse Vincent (Fujifilm, Tokyo, Japan), for our measurements [21]. We measured radiographic parameters, such as lumbar Cobb angle, LIV tilting angle, disc angulation just below the LIV, and vertebral body rotation at the LIV. Radiographic parameters were evaluated at 1st erect, 1 year, 2 years, and the final follow-up. After the radiographic parameters were measured, the factors contributing to the deterioration of the lumbar Cobb angle at the final observation were investigated using multivariate logistic regression analysis.

### 2.4. Statistical Analysis

Data are shown as mean ± standard deviation. Statistical differences between Lenke 1 or 2B and 1 or 2C groups were evaluated using the Mann–Whitney U test and the unpaired *t*-test. The radiographical parameters were also analyzed with the Mann–Whitney U test and unpaired *t*-tests. Multivariate logistic regression and the above statistical analyses were performed using commercially available analysis software (JMP^®^ version 9, SAS Institute Inc., Cary, NC, USA). A *p*-value less than 0.05 was considered statistically significant.

## 3. Results

### 3.1. Basic Characteristics of Patients with AIS

The preoperative lumbar Cobb angle showed significant differences in the Lenke 1 or 2B group and 1 or 2C group (Table 1). The preoperative Cobb angles of the main thoracic (MT) curve in the Lenke B and C groups were 50.6° and 46.8°, respectively (Table 1). All of these patients were evaluated for lumbar Cobb angle, LIV disc angulation, and tilting angle with X-ray imaging, and apical-thoracolumbar/lumbar-vertebral rotation was evaluated with CT imaging.

### 3.2. Thoracolumbar/Lumbar Cobb Angle Before and After Surgery

**Figure 3:**
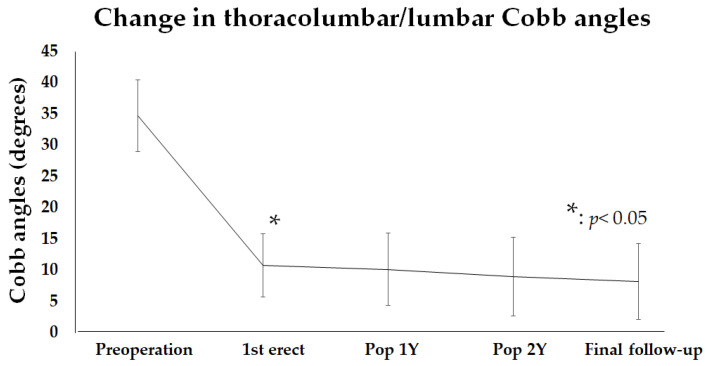
The change in the thoracolumbar/lumbar Cobb angles from preoperation to the final follow-up. The thoracolumbar/lumbar Cobb angle was significantly improved after surgery. The improvement continued until the final follow-up. * indicates clinical significance.

Postoperative changes in thoracolumbar/lumbar Cobb angle were measured until the final follow-up. The average preoperative Cobb angle was 35.1 ± 4.2 degrees, but it improved to about 10 degrees in the postoperative period (1st erect) and finally to about 8.2 ± 6.1 degrees (Figure 3). The thoracolumbar/lumbar curve improved significantly (*p* < 0.01) at 1st erect compared to preoperatively, and then showed a gradual improvement until the final follow-up.

### 3.3. Analysis of Changing in Intervertebral Disc Angulation Below LIV

**Figure 4:**
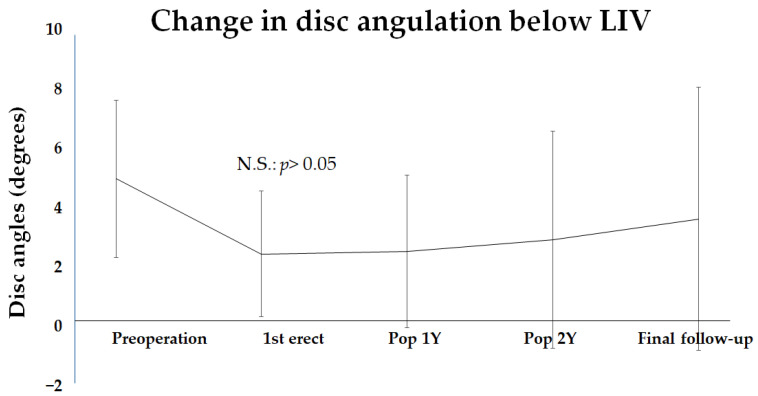
The change in intervertebral disc angulation below the lowest instrumented vertebra (LIV) from preoperation to the final follow-up period. The change in the disc angulation showed no significant postoperative improvement. N.S. indicates nothing significance.

Next, intervertebral disc angulation at the LIV was examined. The mean preoperative disc angulation at the LIV was 5.1 ± 2.8 degrees, which improved to 2.5 ± 2.2 degrees at 1st erect and 3.7 ± 4.6 degrees at the final follow-up (Figure 4). Overall, there was a trend toward improvement, but the variability was large and no clear significant difference was observed.

### 3.4. An Analysis of Changing in LIV Tilting Angle

**Figure 5:**
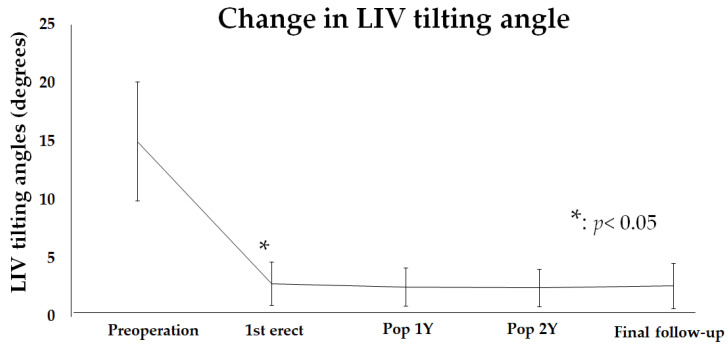
The change in the LIV tilting angle. The LIV tilting angle was improved after surgery. The improvement continued until the final follow-up. * indicates clinical significance.

Furthermore, intervertebral tilting at the LIV was examined. The mean preoperative tilting angle at the LIV was 15.0 ± 5.1 degrees, which significantly improved to 2.8 ± 1.8 degrees at 1st erect (*p* < 0.05) and 2.6 ± 1.9 degrees at the final follow-up (Figure 5). The LIV tilting angle was not significant from 1st erect to the final follow-up, but showed a trend of gradual improvement.

### 3.5. An Analysis of Changing in Thoracolumbar/Lumbar Vertebral Rotation at LIV

**Figure 6:**
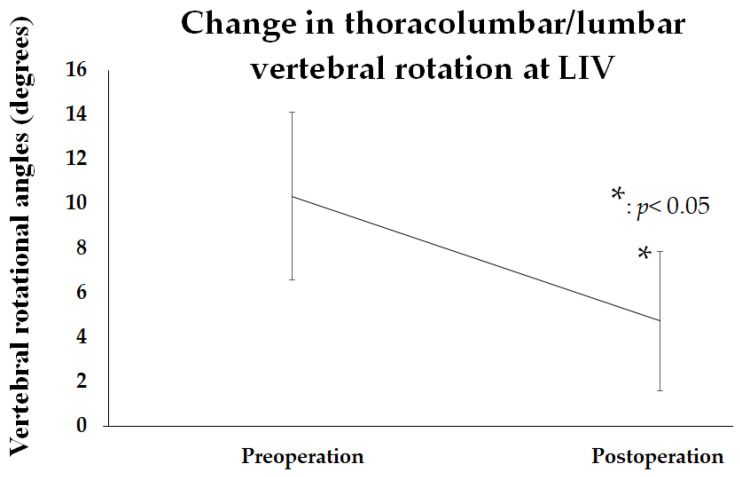
The change in the thoracolumbar/lumbar-vertebral-rotational angle between pre- and postoperation. The amount of postoperative–preoperative change in apical-lumbar-vertebral rotation is shown. * indicates clinical significance.

The thoracolumbar/lumbar vertebral rotational angle at the LIV was measured using the pre- and postoperative CT. The average preoperative angle was 10.3 ± 3.8 degrees, which significantly improved to 4.7 ± 3.1 degrees immediately after surgery (*p* < 0.05) (Figure 6). However, since CT imaging was not performed at the final follow-up, only pre- and postoperative evaluations were performed.

### 3.6. Multivariate Regression Analysis of Deterioration of Thoracolumbar/Lumbar Curve After Surgery

In the thoracolumbar/lumbar Cobb angle at the final follow-up, 9 patients had a final deterioration of 5 degrees or more, and 36 patients had an improvement of less than 5 degrees. A multivariate regression analysis was performed to determine which of the aforementioned variables, such as X-ray parameters and degree of improvement in rotation as measured using CT imaging, were most associated with deterioration in the above-mentioned deteriorated-by-5-degrees-or-more and improved groups. As shown in Table 2, there were significant differences in age, Risser sign, and postoperative Cobb angle (1st erect). The most significant difference was in postoperative Cobb angle (1st erect), suggesting that improvement in the thoracolumbar/lumbar curve immediately after surgery is the most important factor in preventing subsequent deterioration.

### 3.7. Representative Cases in Lenke 1C Using CRT

**Figure 7:**
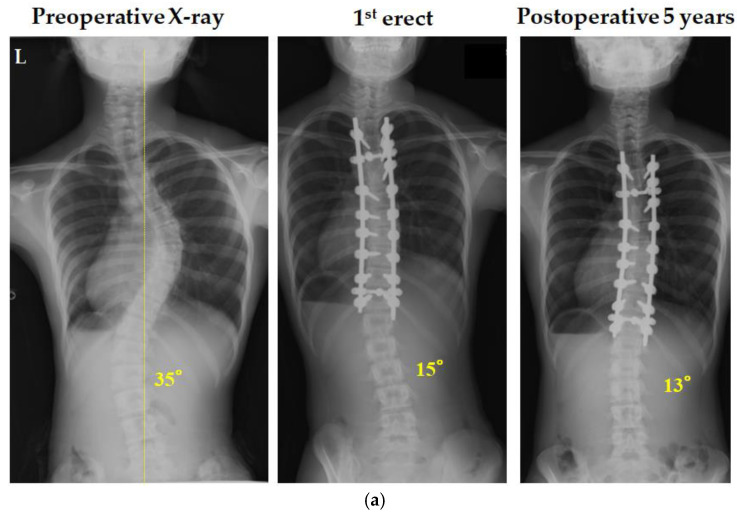

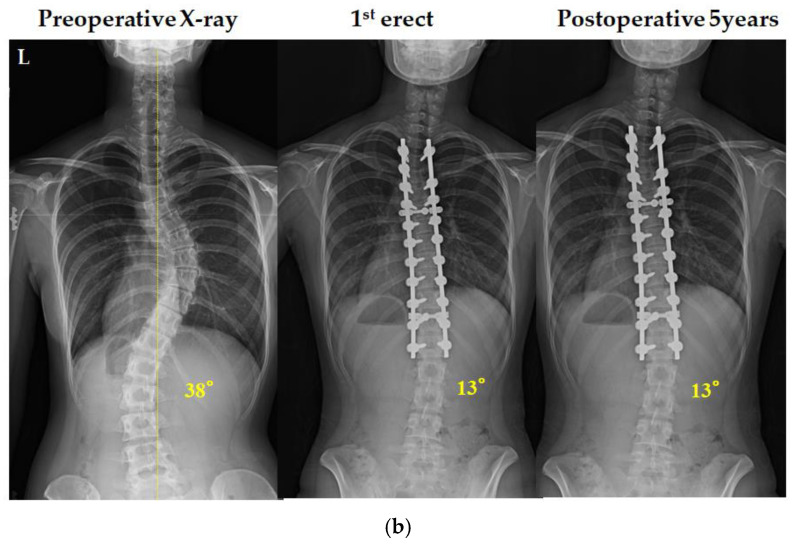
X-rays show a thirteen-year-old girl with Lenke 1C scoliosis (**a**). The preoperative X-ray shows a thoracolumbar/lumbar Cobb angle of 35°. The postoperative X-ray at 1st erect shows a lumbar Cobb angle of 15° after posterior fusion and correction using the CRT technique. The postoperative X-ray at the final follow-up shows a lumbar Cobb angle of 13°. The X-rays show an eighteen-year-old girl with Lenke 1C scoliosis (**b**). The preoperative X-ray shows a thoracolumbar/lumbar Cobb angle of 38°. The postoperative X-ray at 1st erect shows a thoracolumbar/lumbar Cobb angle of 13° after posterior fusion and correction using the CRT technique. The yellow dotted line indicates the central sacral vertical line.

A thirteen-year-old girl with Lenke 1C scoliosis underwent surgery with the CRT technique (Figure 7a). A preoperative X-ray showed a main thoracic and thoracolumbar/lumbar Cobb angle of 48° and 35°, respectively. The lumbar curve improved to 15 degrees in the 1st erect radiograph taken 1 week after surgery, but a slight coronal imbalance remained. A preoperative X-ray of an 18-year-old AIS Lenke 1C curve female is shown in Figure 7b. The preoperative X-ray shows a main thoracic and thoracolumbar/lumbar Cobb angle of 58° and 38°, respectively. Surgery was performed with T3-L1 posterior corrective fixation and CRT on the vertebra below the NV (T11) to correct rotation and improve LIV tilting. The patient had a thoracolumbar/lumbar curve of 38 degrees preoperatively, which improved to 13 degrees immediately after surgery (1st erect) and was eventually maintained.

## 4. Discussion

In the present study, we described the use of CRT to correct the thoracolumbar/lumbar curve in patients with Lenke 1-2 B and C AIS. The CRT technique uses a pedicle screw at the transition from the thoracic spine to the lumbar curve to correct the lumbar curve as much as possible without over-extending fixation.

Our results showed that the Cobb angle of the thoracolumbar/lumbar curve, LIV tilting angle, and vertebral rotational angle at the LIV significantly improved postoperatively and continued to do so until the final follow-up. Multivariate analysis of factors related to the deterioration of the lumbar Cobb angle with clinical and radiographical parameters showed that the most significant and highly relevant effect was the corrected angle of the thoracolumbar/lumbar curve in the immediate postoperative period (1st erect). In other words, the lumbar curve correction in patients with Lenke 1-2 B and C is considered to be less likely to deteriorate if the thoracolumbar/lumbar curve improves immediately after surgery, even without prolonged immobilization. In this CRT, we focused on correcting the vertebral rotation at the transition from the thoracic to the lumbar spine, and at the end, we also corrected the LIV tilting at the same time. The greatest advantage of CRT is that when rotational correction is applied to the lumbar spine below the NV, the corrective force is also transmitted to the lumbar spine curve, albeit indirectly, and the lumbar spinal curve is corrected. It is difficult to measure the corrective force of CRT alone because it is actually a part of a series of surgical procedures. This technique corrects the thoracolumbar/lumbar curve based on the NV of the caudal side of the thoracic curvature. The CRT technique is basically applied to the Lenke 1-2 B or C curve, but may also be applicable to Lenke 3, 4, and 6.

On the other hand, considering spontaneous correction, the lumbar curve shows a slight improvement at 5 years after the surgery, although there is no trend of improvement. Similar changes were observed for the LIV tilting. Previous studies on spontaneous lumbar curve correction and distal adding-on have indicated that they are associated with flexibility of the lumbar curve [23], percent thoracic curve correction, leveling of the LIV [23,24], and restoring thoracolumbar kyphosis [3]. In addition, the preoperative thoracolumbar/lumbar Cobb angle in the supine position under traction as a preoperative radiographic factor correlated most strongly with the spontaneous correction of the TL/L curve Cobb angle after selective thoracic fusion [25]. Furthermore, we did not evaluate the relationship between the rate of correction of the thoracic curve and the thoracolumbar/lumbar curve because the correction of this series was focused on correcting the thoracolumbar/lumbar curve.

Considering the selection of the LIV, the following conditions are reported to be desirable for selective thoracic fusion: a lumbar Cobb angle less than 26°, a coronal balance less than 2 cm, a deformity flexibility index less than 4, a lumbar spine correction greater than 37%, and a trunk shift less than 1.5 cm [26]. Complications of selective spinal fusion include junctional kyphosis, coronal imbalance, distal adding-on, and revision surgery [27]. In addition, it has been reported that Lenke 1C patients are prone to postoperative trunk shift. The reason may be related to the choice of the LIV and the ratio between the Cobb angles of the thoracic and lumbar curvatures [28]. It has also been reported that the preoperative lumbosacral takeoff angle may be a useful predictor of the thoracolumbar/lumbar Cobb angle after selective thoracic fusion surgery [29].

In our case, the patient also displayed a trunk shift to the left preoperatively as shown in Figure 7a. The X-ray of the 1st erect also showed the same leftward decompensated position, but the final follow-up examination at 5 years after surgery showed improvement. This suggests that 2 years postoperatively may not be sufficient time to evaluate the spontaneous correction of the thoracolumbar/lumbar curve because of the time required for bone remodeling and the reestablishment of spinal balance. Long-term follow-up is considered important to evaluate the spontaneous correction of the thoracolumbar/lumbar curve.

There are several limitations to this study. First, as mentioned above, we were not able to evaluate the effect of CRT alone on correction. Because CRT is performed as part of a surgical procedure, it would be very difficult to determine the effect of CRT alone on correction. Second, the relationship between the rate of correction of the thoracic spine curve and the effect of correction of the thoracolumbar/lumbar curve was not evaluated. Third, the Lenke 1-2 B and C curves were evaluated together. However, the present report focuses on the correction of the thoracolumbar/lumbar curve with CRT, and the Lenke 1-2 B and C curves were selected for the needs of this procedure. Fourth, the sagittal profile of the thoracolumbar/lumbar spine was not evaluated in this case. Analysis of the sagittal of the whole spine, including the pelvis, is expected to be the subject of future research. Fifth, we have not investigated physical activity tests such as the Oswestry Disability Index before and after surgery. Therefore, it is difficult to accurately assess physical activity.

## 5. Conclusions

The CRT technique with the LIV selected as SV + 1 showed a significant improvement of the thoracolumbar/lumbar curvature and LIV tilting angle from the postoperative 1st erect period to the final follow-up. Thoracolumbar/lumbar vertebral rotation also showed significant postoperative improvement with CRT. The postoperative thoracolumbar/lumbar Cobb angle (1st erect) was the factor most significantly associated with the deterioration of the thoracolumbar/lumbar curvature after surgery. Subsequent rotational correction of the thoracolumbar/lumbar curvature is likely to prevent the deterioration of the thoracolumbar/lumbar Cobb after surgery.

## Figures and Tables

**Table 1:** The basic characteristics of patients with AIS.

Title	1 or 2B (*n* = 28)	1 or 2C (*n* = 17)	Overall (*n* = 45)	*p*-Value *
Height (cm)	156.1 ± 8.9	155.1 ± 5.2	155.8 ± 7.4	0.81
Weight (kg)	47.6 ± 4.1	45.1 ± 4.2	46.5 ± 6.0	0.68
Age (years)	16.0 ± 4.5	13.6 ± 1.2	15.1 ± 3.2	0.51
Main Thoracic Cobb angle (degrees)	50.6 ± 8.6	46.8 ± 3.4	50.0 ± 8.5	0.08
Thoracolumbar/Lumbar Cobb angle (degrees)	33.5 ± 5.4	37.6 ± 3.6	35.1 ± 4.2	*p* < 0.05
Risser’s sign	3.9 ± 1.7	4.2 ± 0.4	3.9 ± 1.1	0.14
Fixed intervertebral space	10.0 ± 1.5	10.2 ± 1.9	10.0 ± 1.6	0.78

* Statistical analysis was performed to compare the 1 or 2B with the 1 or 2C group using an unpaired *t*-test.

**Table 2:** Multivariate logistic regression analysis.

Title	Odds	*p*-Value
Age	1.2	*p* < 0.01
Risser’s sign	1.4	*p* < 0.05
Postoperative thoracolumbar/lumbar Cobb angle (1st erect)	5.3	*p* < 0.0001

## Data Availability

The data presented in this study are available if requested by the corresponding author.
